# eRAPID electronic patient self-Reporting of Adverse-events: Patient Information and aDvice: a pilot study protocol in pelvic radiotherapy

**DOI:** 10.1186/s40814-018-0304-6

**Published:** 2018-06-05

**Authors:** Patricia Holch, Simon Pini, Ann M. Henry, Susan Davidson, Jacki Routledge, Julia Brown, Kate Absolom, Alexandra Gilbert, Kevin Franks, Claire Hulme, Carolyn Morris, Galina Velikova, on behalf of the eRAPID radiotherapy work group, G. Velikova, J. Blazeby, J. Brown, S. Davidson, A. Gilbert, P. Holch, S. Pini, A. Henry, K. Franks, A. Choudry, C. Harley, J. Routledge, K. Jenner, H. Richards, C. Morris

**Affiliations:** 1 Department of Psychology, School of Social Sciences, Leeds Beckett University, Calverley Building, Room CL 815 City Campus, Leeds, LS1 9HE UK; 2 Section of Patient-Centred Outcomes Research, Patient Reported Outcomes Group, Leeds Institute of Cancer Studies and Pathology, University of Leeds, Bexley Wing, St James’s Hospital, Beckett street, Leeds, LS9 7TF UK; 3 Leeds Teaching Hospitals NHS trust, St James’s Institute of Oncology, Bexley Wing, St James’s Hospital, Beckett Street, Leeds, LS9 7TF UK; 4 The Christie NHS Foundation Trust, 550 Wilmslow Rd, Manchester, M20 4BX UK; 5 Leeds Institute of Clinical Trials Research, University of Leeds, Leeds, LS2 9NL UK; 6 Academic Unit of Health Economics, Leeds Institute of Health Sciences, University of Leeds, Leeds, LS2 9NL UK; 7 Patient Reported Outcomes Group, Leeds Institute of Cancer Studies and Pathology, University of Leeds, Bexley Wing, St James’s Hospital, Beckett street, Leeds, LS9 7TF UK; 8 National Cancer Research Institute Consumer forum, Angel Building, 407 St John Street, London, EC1V 4AD UK

**Keywords:** Cancer, Adverse events, Patient-reported outcome measures (PROMs), Patient-reported outcomes (PROs), Electronic, Electronic health records, Internet, Intervention, Self-management, Radiotherapy, Chemoradiotherapy

## Abstract

**Background:**

An estimated 17,000 patients are treated annually in the UK with radical radiotherapy (RT) for pelvic cancer. New treatment approaches in RT have increased survivorship and changed the subjective toxicity profile for patients who experience acute and long-term pelvic-related adverse events (AE). Multi-disciplinary follow-up creates difficulty for monitoring and responding to these events during treatment and beyond. Originally developed for use in systemic oncology therapy eRAPID (electronic patient self-Reporting of Adverse-events: Patient Information and aDvice) is an online system for patients to report AEs from home. eRAPID enables patient data to be integrated into the electronic patient records for use in clinical practice, provides patient management advice for mild and moderate AE and advice to contact the hospital for severe AE. The system has now been developed for pelvic RT patients, and we aim to test the intervention in a pilot study with staff and patients to inform a future randomised controlled trial (RCT).

**Methods:**

Eligible patients are those attending St James’s University hospital cancer centre and The Christie Hospital Manchester undergoing pelvic radiotherapy+/−chemotherapy/hormonotherapy for prostate, lower gastrointestinal and gynaecological cancers. A prospective 1:1 randomised (intervention or usual care) parallel group design with repeated measures and mixed methods will be employed. We aim to recruit 168 patients following recommendations for sample size estimates for pilot studies. Participants using eRAPID will report AE (at least weekly) from home weekly for 6 weeks and 6 weeks post-treatment (12-week total) then at 18 and 24 weeks. Hospital staff will review eRAPID reports and use information during consultations. Notifications will be sent to the relevant clinical team when severe symptoms are reported. We will measure patient-reported outcomes using validated questionnaires (Functional Assessment in Cancer Therapy Scale-General (FACT-G), European Organisation for Research and Treatment of Cancer Core Quality of Life questionnaire (EORTC-QLQ-C30), process of care impact (hospital records of patient contacts and admissions) and economic variables (EQ5D-5L, patient use of resources)). Staff and patient experiences will be explored via semi-structured interviews.

**Discussion:**

The objectives are to establish feasibility, recruitment, integrity of the system and attrition rates, determine effect sizes and aid selection of the primary outcome measure for a future RCT. We will also refine the intervention by exploring staff and patient views. The overall goal of this complex intervention is to improve the safe delivery of cancer treatments, enhance patient care and standardise documentation of AE within the clinical datasets.

**Trial registration:**

ClinicalTrials.gov NCT02747264.

**Electronic supplementary material:**

The online version of this article (10.1186/s40814-018-0304-6) contains supplementary material, which is available to authorized users.

## Background

An estimated 17,000 patients are treated annually in the UK with radical radiotherapy (RT) for pelvic cancer [1]. New treatment approaches such as intensity-modulated radiotherapy (IMRT), image-guided radiotherapy (IGRT), fractionated delivery [2] and concomitant chemoradiotherapy (chemoradiotherapy (CRT)) have changed the subjective toxicity profile of radiotherapy (RT). Patients survive longer but suffer acute and long-term pelvic-related AE. Multi-disciplinary follow-up creates difficulty for monitoring and responding to these events during treatment and beyond [3]. In order to support patients and fully understand the effects of new treatments, improved and more timely documentation of acute, intermediate and late AE is essential [4].

Prostate patients with localised cancer have good long-term prognosis but endure the long-term effects of pelvic RT [5]. In both cervical and ano-rectal cancer CRT improves outcomes at the expense of additional chemotherapy specific toxicities and the late bowel, urinary and sexual side effects from RT [6, 7]. Overall, for patients treated with pelvic RT 59% women and 45% men will have long-term gastrointestinal (GI) side effects, e.g. bowel urgency [8]. Long-term genitourinary (GU) side effects (e.g. urinary urgency) are experienced by 49% women and 46% men, and 24% women and 53% men state their ability to have a sexual relationship is affected [8].

Assessment and documentation of RT-related AE is generally inconsistent; therefore, improving the systematic and consistent recording of AE would inform strategies to reduce the impact of AEs on patients, clinicians and the health service [4, 6]. Late effects of pelvic RT only fully develop and affect patients’ months/years post-treatment, when centralised follow-up in specialised RT clinics is often infrequent. A cost-effective model to allow remote measurement of RT-AE would help patients to get appropriate specialist advice and support when late toxicity emerges. Systematic data collection would also allow comparisons and evaluation of the benefits of new RT approaches.

In RT, AE are generally reported by clinicians using the RTOG scale for acute AE and the Late Effects on Normal Tissues–Subjective, Objective, Management and Analytic (LENT-SOMA) [9]. Scale, which has been incorporated into the National Cancer Institute’s (NCI) Common Terminology Criteria for Adverse Events (CTCAE v.3), now superseded by v.4 [10]. However, traditionally clinician-reported AEs are heavily focussed on safety and the rare more severe grades of toxicity.

### Patient-reported outcomes (PROs)

Asking patients to report their own symptoms via patient-reported outcomes (PROs) has proven extremely acceptable to patients in the oncology clinic setting [10–13]. Reviews suggest they improve symptom/function monitoring, physician patient communication, decision-making [14–19], timing and accuracy of symptom reporting [20]. PROs have been increasingly recommended by the United States Food and Drug Administration (FDA) [21] and here in the UK in the 2008 Darzi report [22]. The Department of Health (DOH) subsequently produced guidelines to aid their implementation [23], and we now have some nationwide use of PROs in the NHS [24]. In terms of monitoring toxicity, the CTCAE scale has recently been adapted for patients to self-report (NCI-PRO-CTCAE) [25, 26]. These items have concordance with nurse-evaluated AE [27] and similar items created for self-report correlate with quality of life (QOL) measures [28]. Increasingly, patients are being asked to monitor their own health status and self-manage symptoms. In response to this, we have developed the eRAPID system to improve the detection, and management of AE in cancer patients by providing the means by which patients can report symptoms from home [29, 30].

### Electronic PROs (ePROs)

The use of ePROs in health research has risen exponentially during the past 20 years for a number of conditions including cancer [31, 32]. Patients have shown they can routinely complete PROs electronically, [11] and paper and electronic completions are comparable [33]. Further, RT patients and staff said they would find an internet PRO reporting tool acceptable. Clinicians thought this would improve their consultations with having graphical representation of the PRO data [34]. It is also clear that patients are willing and able to use PROs from home internet or mobile devices [35, 36].

Examples of successful implementation of electronic symptom reporting in oncology clinical practice include PatientViewpoint [37], a flexible PRO platform, the symptom tracking and reporting system (STAR) for patients to report chemotherapy AE [35], and the Tell Us™ [38] system for advanced cancer patients in hospices undergoing palliative care (all in the USA). In Canada, they have developed an interactive online system (ISAAC) [39] and in the UK the ASyMS mobile phone system [40]. Electronic patient-reported outcome systems have proven very acceptable even for patients coping with extreme symptom burden and reduced QOL; indeed, a mean monthly PROM completion rate of 83% at 34 weeks was achieved with patients receiving cancer treatment [41]. In RT, the VisionTree Optimal Care online reporting tool has proven acceptable to patients and increased QOL completion compliance to 90% compared to paper completion of 50% [42].

### eRAPID development

In Leeds, we recently commissioned the development of a web-based questionnaire builder system called QTool (built by X-Lab, a private software company and funded by Macmillan Cancer Support and Cancer Research UK) (www.epocs.leeds.ac.uk). QTool Version1 was used in a large prospective study of cancer survivors [43]; however, the PROs were not viewed by clinicians. Utilising this technology eRAPID is an online system to support the collection and clinical integration of patients’ symptom/AE reports during cancer treatment [30]. Between 2010 and 2013, the eRAPID developmental work was conducted (funded by a National Institute of Health Programme Development Grant RP-DG-1209-10,031). Developed originally for use in systemic treatment, the system enables patients to report AE via PROs [44] and the questionnaire data is integrated into the electronic patient records (EPR) for clinicians to view. Algorithmic questionnaire scoring [44, 45] generates severity dependent management advice to either call the hospital or self-manage symptoms (accessible via a custom build website). Currently, an RCT in systemic therapy is underway recruiting 500 patients [46]. We have now adapted the system for use in upper gastrointestinal surgery and RT.

### Multi-site eRAPID radiotherapy development

Developmental work was conducted to adapt the eRAPID system for RT in two centres (Leeds and The Christie Hospital Manchester) and for patients undergoing pelvic radiotherapy for urological, gastrointestinal and gynaecological cancer. A rigorous systematic approach was adopted according to the criteria for reporting the development and evaluation of complex interventions in healthcare [47] and the Medical Research Council (MRC) guidelines [48].

In this two-centre study, professional and patient care pathways and the role of eRAPID were explored via audit and interviews with staff, patients and relatives [49–51]. Additionally, we identified the AEs associated with urological, gastrointestinal and gynaecological cancers from patient interviews [50] and via systematic reviews of PRO reporting for these groups [52–54]. In a mapping exercise to matching questionnaire items to common adverse events revealed the best questionnaire coverage for adverse events for all tumour groups were the male and female pelvic questionnaires (MPC and FPC) [55]. For a clinical perspective Delphi (consensus methodology) was used with staff to make decisions on the most appropriate items for each AE [56]. This led to the inclusion of additional items from the EPIC (Expanded Prostate Cancer Index) and the EORTC-QLQC-30 and PR 25 (prostate module). In addition, to cover chemotherapy-related AE items were taken from item pool developed for eRAPID (systemic therapy) [44]. See (Additional file 1: Appendix 1) for example of AE items used either as standard, branching (having a prior qualifying question) or as a drop-down (allowing selection of more appropriate options).

The AE questionnaires are scored according to specific algorithms developed in consultation with key staff and patients. Clinically meaningful cut scores for symptom severity were developed through consenus and discussion based methods [57]. The scorning and algorithms allows the generation of severity-dependent management advice for either mild (self-management advice), moderate (self-management and advice to contact the hospital when appropriate) or severe (advice to contact the hospital immediately). The self-management advice for mild level symptoms was developed from local and reputable national resources [58] and uploaded to two designated websites for Leeds and The Christie patients. AE. The online system is now successfully implemented at Leeds Teaching Hospitals NHS Trust (LTHT) and The Christie Hospital Manchester.

### The eRAPID intervention in radiotherapy

**Fig. 1:**
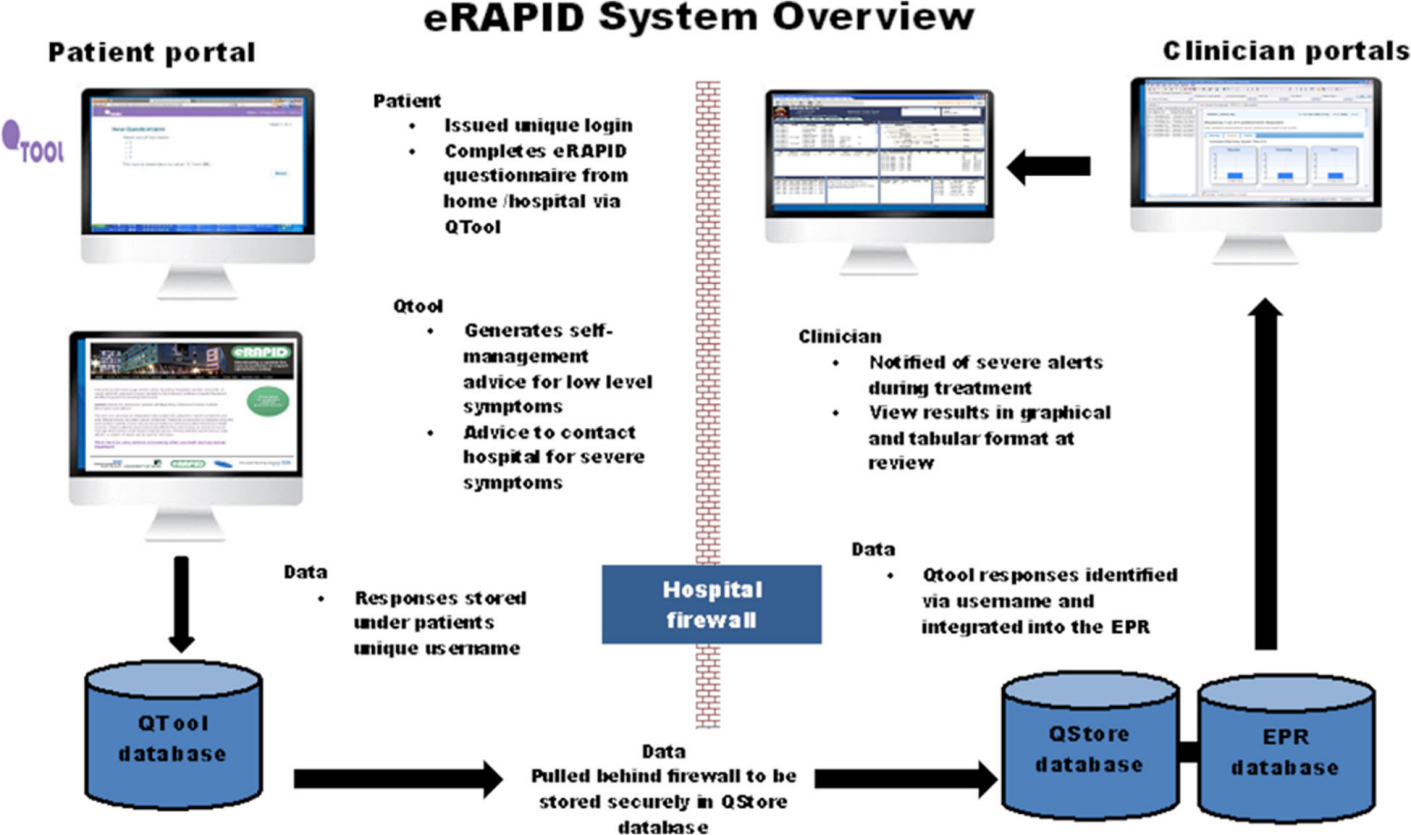
eRAPID system architecture overview

**Fig. 2:**
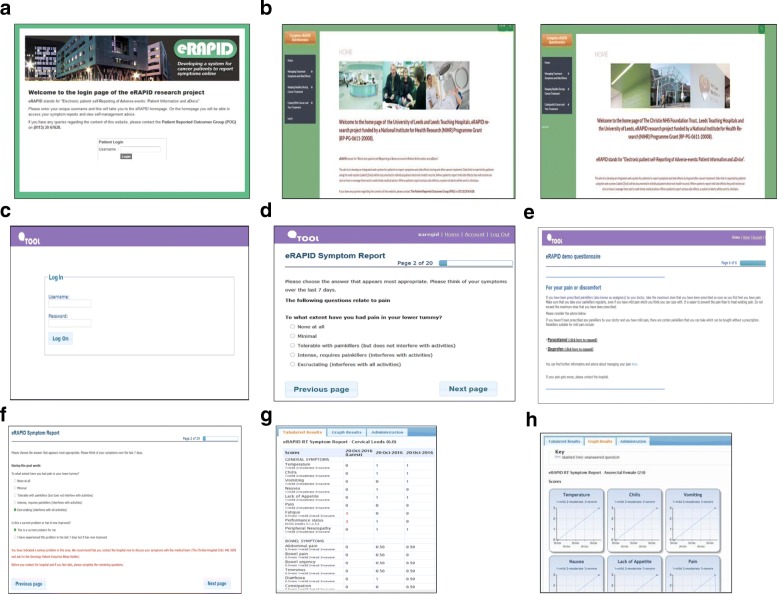
Screenshots of eRAPID intervention Patient login (**a**-**f**) **a** Login eRAPID portal, **b** access eRAPID websites for (QTool) login (Leeds and Manchester), **c** login to online symptom report, **d** complete symptom report, **e** recieve advice to manage low-level symptoms, **f** login to online symptom report. Clinician view of symptom reports in electronic patient record (EPR) (**g** & **h**) **g** symptom reports in EPR (tabular view), **h** symptom reports in EPR (grapical view)

The developmental work led to the eRAPID complex intervention [44, 46] consisting of the following components (see Figs. 1 and 2):

- Patients login to QTool using unique usernames and password and complete the eRAPID symptom questionnaire from home on computers/mobile phones/tablets or using designated kiosks (Leeds). Immediate tailored automated advice, calculated from a series of algorithms, is generated.
- If severe symptoms are reported, patients are advised to contact the hospital.
- For mild/moderate problem information about self-managing, these issues are provided via brief instructions in QTool and hyperlinks to more detailed advice on the eRAPID RT patient websites tailored to Leeds and The Christie local information.
- The patient-reported data is immediately available for staff to review in the individuals’ EPR in Leeds Teaching Hospitals NHS Trust (PPM), The Christie Clinical Web Portal (CWP).
- Notifications for severe symptom reports are sent directly to staff via email. Clinicians can then login to PPM and CWP and view the patients’ symptom reports and take appropriate action where needed.

### Aims and objectives

We aimed to conduct the initial pilot study of the eRAPID system in radiotherapy from the perspective of cancer patients and health professionals. The objectives are to establish feasibility and determine effect sizes of the potential benefits of the intervention for a future RCT.

The specific aims are to:

- Establish recruitment and attrition rates
- Establish adherence to symptom reporting and record the number of calls made to the hospital by patients
- Test the integrity of the study protocol and data collection forms
- Test the integrity of information systems designed to collect and collate the data
- Aid selection of the most appropriate primary outcome measure and determine effect size
- Refine the intervention by exploring patient and staff views.

## Methods

### Ethics

The study procedures below reflect the protocol version 1.3 as approved by the National Research Ethics Service (now part of the Health Research Authority). The approval was from Yorkshire & The Humber Leeds East Research Ethics Committee on 13 September 2016 (REC reference 16/YH/0371). Local approvals from the Leeds Teaching Hospitals NHS Trust and The Christie Research and Innovation Department were also obtained. Protocol amendments will be communicated via written statements and verbally reiterated where appropriate to the funder, trial teams, clinicians, steering and project management groups and trial registries.

### Patient sample

The sample will include two groups of participants at both The Christie Hospital Manchester (C) and Leeds Teaching Hospitals NHS Trust (L):

1. Patients receiving radical RT for prostate cancer (including radiotherapy for prostate cancer +/− hormonotherapy+/− brachytherapy boost) (C & L).
2. Patients receiving pelvic chemoradiotherapy including cervical (C & L), anal (C & L), rectal (L), vaginal (L), vulval (C) and endometrial (C).

### Inclusion criteria

Patients will be eligible if

- They have a diagnosis of (1) prostate cancer requiring radical radiotherapy treatment (including radiotherapy +/− brachytherapy boost) or (2) anal, rectal, cervical, endometrial, vaginal or vulval cancer requiring chemoradiotherapy.
- Aged 18 years or over.
- Attending St James’ University Hospital or The Christie Hospital Manchester.
- Able and willing to give informed consent.
- Read and understand English have access to the internet at home.

### Exclusion criteria

Patients were excluded if taking part in other clinical trials involving the completion of extensive PRO or QOL measures or exhibiting overt psychopathology/cognitive dysfunction.

### Patient pathways

The chemoradiotherapy pathways (cervical, anal, rectal, endometrial, vaginal and vulval patients) are very similar at both centres. However, the cervical patients in Leeds get a brachytherapy (BT) boost at 5 weeks and The Christie patients undergo this at 6 weeks. At Leeds after completion of radiotherapy, rectal patients (post-surgery) are followed up by the surgeons. Patients will be encouraged to complete AE reporting for radiotherapy toxicity but will be informed the surgical team will not be looking at the patient-reported data at 12 and 24 weeks. However, outcome measures will still be collected at 24 weeks. The radical prostate treatment pathway is similar at both centres (The Christie and Leeds); however, The Christie offers a 6-week follow-up via the telephone. All treatment pathways were mapped, and a detailed flow charts were made including timelines, toxicities, treatment visits and health personnel involved [59].

### Comparability between centres

Although the Leeds and The Christie treatment schedules differ slightly for each of the cancer sites, the treatment volumes will be comparable. Similarly, the timescales of toxicity will also be comparable, e.g. acute bowel effects from pelvic RT usually start from 8th to 9th fraction, i.e. at end of the second week.

### Design

**Fig. 3:**
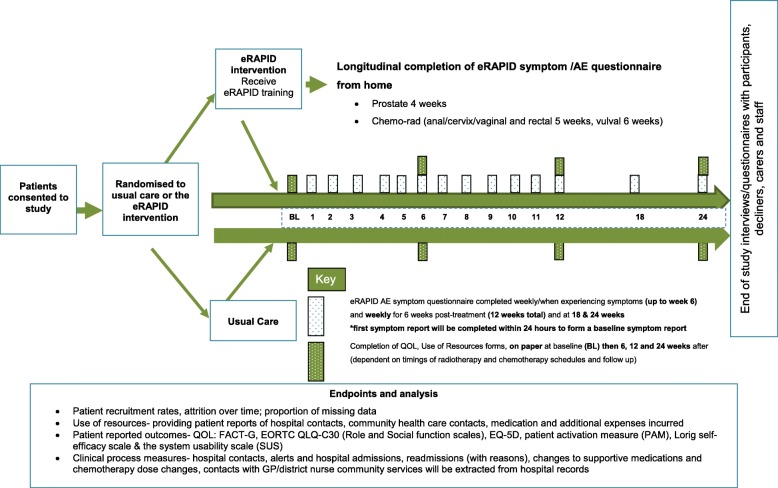
Trial flow diagram for the eRAPID radiotherapy feasibility pilot study

This study is conducted at two centres (The Christie (C) and Leeds (L) employing a prospective randomised two-arm parallel group design with repeated measures and mixed methods. Participants will be randomised to either the intervention arm (eRAPID RT plus usual care) or the control arm (usual care) with 1:1 allocation). See Fig. 3 for the Trial Flow diagram.

### eRAPID intervention

Only patients randomised to the intervention will complete the eRAPID online symptom/AE report within 24 h of study entry (forming the baseline symptom report) and then weekly (or when experiencing symptoms) for 12 weeks (6 weeks during treatment and 6 weeks post-treatment) and at 18 and 24 weeks. Patients in the eRAPID intervention arm will also complete self-efficacy and patient activation measures and a questionnaire to assess their ability to use the online system (system usability scale).

All patients (either eRAPID or usual care) complete baseline measures (pre-randomisation) on paper and at 6, 12 and 24 weeks.

The timings of online completion and the outcome measures were chosen based on the RT schedules and the follow-up pathways, aiming to find a schedule of completions that fits but allows for the slight differences between the two centres.

### Usual cancer care

Patients are provided with verbal and written information on treatment benefits and expected AEs, and instructions on how to manage AE and contact the hospital including out-of-hours. They have a nurse/clinical oncologist/radiographer assessment before starting treatment and are given information on how to manage AE and out-of-hours contact numbers.

### Recruitment

Patients will be recruited from RT departments, outpatient clinics and day-case wards at Leeds Teaching Hospital NHS Trust and at The Christie Hospital Manchester (CHM). Eligible patients will be identified by screening of clinic, in-patient or day-case lists by the clinical or administration staff. Consultants responsible for the care of patients are informed via letter or email.

After introduction from clinical staff, a researcher (who has received Good Clinical Practice training (GCP) will explain the study, provide an information sheet (Additional file 1: Appendix 2) and obtain consent (Additional file 1: Appendix 3) at this or the patient’s next visit. Participants are free to withdraw from the trial at any time without giving reasons and without prejudicing any further treatment. Reassurance is given that anonymity will be maintained (patients are issued a study number), and data collected will be treated in the strictest confidence and held in accordance with the Data Protection Act 1998.

### Randomisation

Participants are randomised with 1:1 allocation to intervention and control groups, performed centrally at the University of Leeds Clinical Trials Research Unit (CTRU) using the 24-h telephone system. Patients randomised to the intervention arm receive training in using the eRAPID system.

**Fig. 4:**
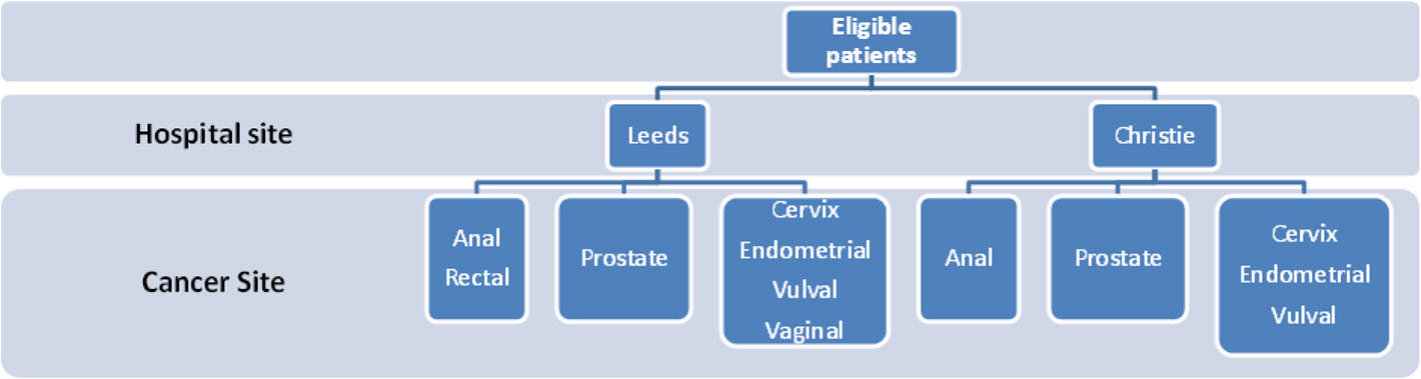
Stratification factors for eligible patients by hospital and cancer site

Patients are already stratified by cancer centre (Leeds or Manchester) and will be further stratified by cancer site, e.g. prostate or cancer requiring chemo RT (cervix, anal, rectal, vaginal, endometrial and vulval). A diagrammatic representation of the stratification factors is presented in Fig. 4.

### Training

#### Staff

Staff are trained by the researchers reinforced by a user-manual providing scenarios of an ‘example’ patient and how to interact, interpret and access the PRO data in the EPR and incorporate this data into their consultations. It is stressed that PRO data should be seen as a supplementary resource to aid management decisions. We will explore and reassure staff that using eRAPID will not extend consultation times.

#### Patient

Researchers demonstrate how to use eRAPID system and provide a unique user name and password to access the system, on an eRAPID ‘postcard’. Participants are given step-by-step user guide and if encounter problems (e.g. lost user names/password or problems logging on) are advised to contact the research team. Prompts to complete the eRAPID questionnaire can be sent via email or text message.

### Sample size and recruitment rate

Thirty patients per arm for each treatment type will provide valuable information on outcomes [60] (Tables 1 and 2) Allowing for 30% overall attrition, we aim to recruit a minimum of 42 patients per-arm (*n* = 84 for each treatment type) at a rate of around 15 patients per month for approximately 15 months giving an overall total of 168 patients, with a 24-week follow-up.

**Table 1:** eRAPID RT study questionnaires and Case Report Forms (CRFs): researcher completed

Questionnaire/CRF	Time point for completion
Eligibility checklist	Baseline
Comorbidity form	End of study
Consent form	Baseline
Registration/randomisation form	Baseline
Baseline: clinical data form	End of study
Clinical process measures Number of hospital contacts Number of alerts and hospital admissions Readmissions (with reasons) Clinician records of CTCAE and RTOG Changes to supportive medications and RT and/or chemotherapy dose changes Contacts with GP/community services Safety monitoring acute admissions, cumulative deaths	Collected during a course of RT treatment and if appropriate chemotherapy cycle (from hospital records and via brief patient survey at routine clinic appointments for interim assessment of clinical contacts)
IT system functioning Telephone log of phone calls from patients Record of unscheduled server down time	Throughout the duration of the study
Death and withdrawal form	At point of death/withdrawal

**Table 2:** eRAPID RT study questionnaires and Case Report Forms (CRFs): participant completed

Questionnaire/CRF	Description of time points for completion	Baseline	3 weeks	6 weeks	9 weeks	12 weeks	18 weeks	24 weeks
Consent form	Baseline	X						
Baseline: patient sociodemographic data form	Baseline	X						
Baseline: computer usage questionnaire	Baseline	X						
Patient-reported eRAPID symptom questionnaire (eRAPID intervention arm only)	Within 24 h of study entry and weekly during treatment and 6 weeks afterwards (12 weeks total). Then at 18 and 24 weeks.	X weekly	X weekly	X weekly	X weekly	X weekly	X (except rectal)	X (except rectal)
Patient Outcome Measures FACT-G EORTC-QLQ-C30 EQ-5D-5 L	Baseline, 6, 12 and 24 weeks	X		X		X		X
Self-efficacy, Lorig 6-item	Baseline and 12 weeks	X				X		
Patient Activation Measure (PAM)	Baseline and 12 weeks	X				X		
Use of resources form	6, 12 and 24 weeks			X		X		X
System Usability Scale	24 weeks (eRAPID intervention arm only)							X
Participant withdrawal feedback form	At point of withdrawal							
End of study patient questionnaire	24 weeks							X
Clinician questionnaires								
Clinician eRAPID feedback form (eRAPID intervention arm only)	At routine hospital appointments throughout the study		X	X	X	X	X (except rectal)	X (except rectal)
Clinician record of CTCAE	To be completed by clinician at the follow-up appointment 4–6 weeks after completion of treatment (exact time-point will be different for different disease groups)							

Statistical analysis of the recruitment and attrition rates, clinical process measures and patient outcome data is the responsibility of the CTRU Trial Statistician under the supervision of the Supervising Statistician. A full statistical analysis plan has been written by the CTRU Trial Statistician. A health economic data analysis will be undertaken by a health economist from the academic Unit of Health Economics (AUHE), based on the health economics analysis plan (HEAP). Interview data will be processed by the research teams in the two participating centres. All analyses will be conducted according to the intention to treat (ITT) principle using SAS statistical software.

## Data collection and management

An overview of data to be collected during the study is outlined in Tables 1 and 2.

### Baseline assessments

All participants complete baseline questionnaires, including socio-demographics and current computer usage. Clinical baseline data will be obtained from participants’ medical notes including sex, age, diagnosis, co-morbidities, smoking status and planned treatment. Patients will then be randomised to receive either usual care or the eRAPID intervention (in addition to usual care). Participants receiving the eRAPID intervention will also be asked whether they want to receive reminders via email or text message to complete their weekly online AE reports. Patients randomised to the eRAPID arm will complete the first online report within 24 h as a baseline symptom report.

### On-going online symptom reporting from home

Patient in the eRAPID arm will complete the AE symptom report from home on a weekly basis during treatment (up to 6 weeks) and for 6 weeks after (12 weeks in total).

### Patient outcome measures

The following measures will be used to enable comparison between participants in the control and eRAPID intervention arms and will be collected on paper. Measures will be completed on paper at baseline then 6, 12 and 24 weeks from randomisation. The research team will either provide the measures at routine hospital appointments or post them to participants (and provide pre-paid envelopes for their return).

#### Use of resources form (completed at 6, 12 and 24 weeks)

Resource use will be assessed using a patient questionnaire (detailing contacts with GPs/community services, hospital visits), developed for a trial assessing treatment for chemotherapy-related nausea/vomiting (http://journalslibrary.nihr.ac.uk/hta/hta17260). This has been revised for use in RT from staff and patient input.

#### Quality of life and wellbeing

To assess the quality of life and wellbeing of the study participants, the following validated measures will be used as patient-reported outcomes:

#### EQ-5D-5L [61] (completed at baseline, 6, 12 and 24 weeks)

The EQ-5D is a standardised health outcome measure developed by the EuroQol Group. EQ-5D has been used with a range of health conditions and treatments providing a simple descriptive profile and single index value for health status for use as part of a health economic evaluation. The instrument assesses five dimensions: mobility, self-care, usual activities, pain/discomfort and anxiety/depression with five response levels (ranging from no problems to extreme problems). The instrument also includes a scale to rate health from 0 to 100 (worst-best health you can imagine).

#### Functional Assessment in Cancer Therapy Scale-General [62] (FACT-G, completed at baseline, 6, 12 and 24 weeks)

The FACT-G is a cancer-specific measure widely used in clinical trials. It has four subscales: physical wellbeing, social or family wellbeing, emotional wellbeing, and functional wellbeing. Question responses range from 0 to 4. Higher scores on the questionnaire indicate better HRQL (score range, 0 to 108).

#### EORTC-QLQ-C30 [63] (completed at baseline, 6, 12 and 24 weeks)

The EORTC-QLQ-C30 is a 30-item questionnaire consisting of five functional scales (physical, emotional, cognitive, social, role), three symptom scales (fatigue, pain, nausea/vomiting), a global health-related quality of life scale and six single items (anorexia, insomnia, dyspnoea, diarrhoea, constipation, financial difficulties). Questions are rated on a 4 or 7-point response scale, and overall scale scores are calculated from 0 to 100 with higher scores indicating better quality of life.

#### Self-efficacy and patient engagement in health care

##### Self-Efficacy for Managing Chronic Disease 6-Item Scale [64, 65] (completed at baseline and 12 weeks)

This 6-item scale contains items taken from several self-efficacy scales. It covers several domains common across many chronic diseases such as symptom control, role function, emotional functioning and communicating with physicians.

#### The Patient Activation Measure [66] (PAM, completed at baseline and 12 weeks)

The PAM is a tool for measuring the level of patient engagement in their healthcare. The PAM 13-item scale explores beliefs, knowledge and confidence for engaging in health behaviours. Each item is rated on a four-point scale from strongly disagree to strongly agree, and an overall score from 0 to 100 can be calculated. These scores can be subdivided to categorise people into one of four activation categories ranging from 1-low activation to 4-high activation.

#### Patient satisfaction with the eRAPID technology

##### System Usability Scale [67] (SUS, completed at 24 weeks––eRAPID intervention arm only)

The SUS is a 10-item instrument to assess subjective views of usability of different systems including hardware, software, mobile devices, websites and applications. The 10 items cover the ease of using the system, its complexity and user confidence. Each item is rated from 1 to 5, and a composite score of overall usability can be calculated ranging from 0 to 100.

### Clinical process measures

To assess any association between the eRAPID intervention and improved detection and management of AEs, the data will be collected from hospital triage forms, medical records and hospital databases to record:

- Number of scheduled and unscheduled hospital contacts (admissions, clinic visits, phone calls with staff)
- Changes to supportive medications, radiotherapy regimens and chemotherapy dose changes
- Contacts with GP/district nurse/community services
- Number of clinician alerts generated from eRAPID severe symptom reports and actions taken by staff.

In addition to the above, at routine radiotherapy review, staff will be asked to provide:

- Clinician reports on use of eRAPID patient data during consultations
- Clinician records of CTCAE matching those AE questions completed by patients on the eRAPID questionnaire at routine clinic visits at 4–6 weeks after treatment completion (exact time-point will be different for different disease groups)

### End of study interviews and questionnaires

At the end of the study, a subset of patients and staff will be interviewed and asked about their views of the eRAPID system. All participants will be asked to complete a short end of study questionnaire about their experiences with the eRAPID intervention.

### Participant interviews and questionnaires

At the end of the study, a purposive sample (by gender and age) of up to 5 participants per disease group from the intervention arm will be invited to interview. They will be asked about the relevance and burden of completing routine PROMs throughout the study, and if the eRAPID system had any impact on their care.

Where possible, participants who withdrew will be asked to complete a voluntary brief feedback form to assess potential reasons for withdrawal.

### Staff interviews and questionnaires

Up to five health professionals from each disease group will be interviewed at the end of the study to determine their views of eRAPID, the perceived value and use of patient data in clinical practice, exploring if eRAPID improves the detection, documentation and management of AE and support treatment decision-making. Perceptions of staff training needs and recommendations for improving the system will also be explored.

## Statistical analysis

### Baseline characteristics

Data from the baseline socio-demographic, computer usage and clinical data questionnaires will be tabulated using frequencies and summary statistics for each treatment group and overall.

### Recruitment missing data and attrition

The feasibility of the recruitment strategy will be evaluated by summarising the screening, eligibility and consent processes, including the number of participants involved at each stage. Where available, reasons for ineligibility and non-participation in the study will be summarised. Retention during follow-up, including the number of participant’s withdrawing from the study and the timing of and reasons for withdrawal will be presented. Acceptability and potential conditions for improved acceptability will be explored as part of the patient and staff interviews.

### Clinical process measures

The number of telephone calls to hospital staff and weekly/additional AE reports completed using the eRAPID system will be summarised. Clinician and staff acceptability will be explored during the staff interviews. Initial interviews will determine any anticipated barriers to the use of eRAPID, whereas exit interviews will explore actual issues that prevented staff from incorporating information into routine practice. Clinicians will be asked when and how they made use of the additional information, and how this benefitted them.

### Integrity of the study protocol and data collection forms

Any deviations from the study protocol, such as ineligible patients being registered into the study, will be reported. The time taken to complete the paper and QTool forms and the amount of missing data will be summarised.

### Integrity of the information systems

Descriptive accounts of any issues with server downtime leading to the unavailability of either the QTool questionnaire or patient-reported data in PPM and CWP will be collected in order to evaluate the overall performance and reliability of the IT system. If these are found to be unacceptable, the relevant server hosts will be contacted. Data from the participant phone call logs will be categorised to assess any common problems encountered and to determine if any changes should be made to the eRAPID training session/user guide to support participants more effectively. The System Usability Scale (SUS) will be compared against available SUS scores for other systems.

### eRAPID system performance

Throughout the study, the eRAPID IT systems will be monitored for unscheduled server down time (leading to the unavailability of the QTool questionnaire website, eRAPID website and patient symptom data in PPM and CWP). This is facilitated via a service level agreement with University of Leeds IT services and protected by the NHS firewall. The service provider is committed to providing 99.9% uptime, and no unscheduled downtime has occurred during the current study. Additionally, a log of phone calls to the research team regarding issues/problems surrounding the use of eRAPID will be maintained.

### Selection of patient outcome measures

Patient outcome measures (see Table 2) will be summarised at each time point, and 95% confidence intervals constructed for the differences between treatment arms. In addition, the number of questionnaires completed and the extent of missing data will be presented. To aid selection for a future RCT, any ceiling and/or floor effects from the questionnaires will be noted.

### Health economic data

Patient-generated data based on the use of resources form will be analysed descriptively for reported frequencies of events and missing data. Comparisons will be made between the number of hospital contacts and admissions made by researchers with that provided by patients.

### Qualitative data

Interviews will be recorded and transcribed. Data will be managed by NViVO software and analysed using thematic analysis [68, 69]. To improve reliability and consistency within the coding process, two researchers will independently conduct the analysis before meeting to discuss and resolve inconsistencies. Understanding of the data will be further refined by using constant comparison, contrastive analysis and looking for negative cases.

### Trial monitoring

#### Trial management group (TMG)

The TMG, comprising the Chief Investigator (CI), Patient Reported Outcomes Group (POG) and the Observational and Supportive Team (OST) at The Christie, patient representatives, CTRU team and other key external members of staff involved in the trial will be assigned responsibility for the clinical setup, on-going management, promotion of the trial and for the interpretation and publishing of the results. Specifically, (i) protocol completion; (ii) case report form development; (iii) obtaining approval from the main REC and supporting applications for Site Specific Assessments; (iv) completing cost estimates and project initiation; (v) facilitating the Trial Steering Committee (TSC); (vi) monitoring of screening, recruitment, treatment and follow-up procedures and (vii) auditing consent procedures, data collection, trial endpoint validation and database development.

#### Trial Steering Committee (TSC)

The independent Steering Committee for the overall eRAPID programme will provide supervision of the RT trial, in particular trial progress, adherence to protocol, participant safety and consideration of new information. In line with NIHR guidance, the TSC includes an Independent Chair, three other independent members including a patient representative. The Committee meets in person annually as a minimum, with six monthly meeting via teleconference. The TSC receives the TMG reports and reports to the Sponsor and the funder (NIHR). The CI and other members of the Trial Management Group (TMG) may attend the TSC meetings and present and report progress. In addition, representatives of the NIHR and of the Sponsor are invited to all meetings and receive minutes.

#### Data monitoring (DMEC)

Routine data collection will be monitored for quality and completeness in Leeds by POG and in Manchester by the OST, using verification, validation and checking processes. Missing data, except individual data items collected from weekly symptom questionnaires, will be chased until they are received or confirmed as not available.

Any serious concerns about data will be referred to the main trial data monitoring committee for systemic therapy which is independent of the sponsor and competing interests [45].

#### Safety monitoring plan

Monitoring acute admissions, cumulative deaths, cause of death and other detrimental effects to patients will assess the possibility of severe AEs being missed as a result of the intervention. These will be reviewed monthly by the CI, reported at each meeting of the TMG, and a 3-monthly report will be submitted for review to the TSC. In addition, the CI will review on a monthly basis patient-reported AE, note any serious reactions and report where appropriate via the online Yellow card scheme https://yellowcard.mhra.gov.uk.

### Modifications

Research ethics committee (REC) will be sought for modifications to the protocol, and any approved changes will be communicated in written form (and reiterated verbally) to all concerned parties including funders, investigators, staff and trial participants registries and regulators.

### Clinical governance

The overall clinical responsibility and welfare of the patients will remain with the individual treating clinicians within each disease group.

### Dissemination policy

The trial is registered with an authorised registry, according to the International Committee of Medical Journal Editors (ICMJE) guidelines, prior to the start of recruitment. Credit for the main results will be given to all those who have collaborated in the trial, through authorship and contributorship. Uniform requirements for submission to medical journals will guide authorship decisions. The CI, relevant research, CTRU and AUHE staff will be named as authors in any publication. In addition, all collaborators will be listed as contributors for the main trial publication, giving details of roles in planning, conducting and reporting the trial. To maintain the scientific integrity of the trial, data will not be released prior to the first publication of the analysis of the primary endpoint, either for trial publication or oral presentation purposes, without the permission of the TSC. In addition, individual collaborators must not publish data concerning their participants directly relevant to the questions posed in the trial until the first publication of the analysis of the primary endpoint. See Abbreviations below.

## Discussion

This paper describes the protocol for the eRAPID feasibility study in patients undergoing pelvic radiotherapy. eRAPID is a complex web-based intervention designed to improve the systematic reporting of AE during cancer treatment and improve patient care and experiences. We aim to determine the acceptability and feasibility from both the perspective of patients undergoing pelvic radiotherapy+/− chemo-radiotherapy and their health professionals to determine effect sizes and select an appropriate outcome measure for a future RCT. We also aim to refine the intervention by exploring patient and staff views.

The immediate severity-related guidance generated from eRAPID on how to manage AE is a unique feature of our system compared to other ePRO web-portals [35, 38]. We hypothesise that eRAPID will ultimately have benefits for patients and staff. For patients, it may enable earlier symptom detection and improved self-management, lead to more timely admissions to manage more serious AE, improve supportive medication use and appropriate health service contacts. For staff, it may reduce the number of contacts, save time spent on enquiring about and recording AEs, focus attention and support decision-making during consultations.

The ultimate success of eRAPID will depend on how patients and staff engage with and appreciate the potential benefits of the system. This pilot study will determine the acceptability and feasibility of the complex intervention for patients and staff and determine any changes required to improve the system prior to evaluation in an RCT. The study is funded as part of 5-year programme and has the potential to provide a cost-effective solution for follow-up for pelvic radiotherapy patients by reducing hospital or primary care contacts. In addition, it will help to create a comprehensive database of RT AE and deliver vital PRO information on treatments.

## Additional file

Additional file 1: Appendix 1. Radiotherapy eRAPID symptom report items. Appendix 2. Patient information sheet. Appendix 3. Patient consent form. (DOCX 50 kb)

## Abbreviations

AE: Adverse events
ASyMS: Advanced Symptom Management System
AUHE: Academic Unit of Health Economics
BT: Brachytherapy––a technique where radioactive therapeutic seeds or rods are implanted into the cancerous area (prostate or cervix)
CHM: The Christie Hospital Manchester
CRT: Concomitant Chemoradiation
CTCAE: Common Terminology Criteria for Adverse Events
CTRU: Clinical Trials Research Unit
CWP: Clinical Web Portal (electronic patient record system at The Christie Manchester)
DMEC: Data Monitoring and Ethics committee
EORTC-QLQ-C30: European Organisation for Research and Treatment of Cancer Core Quality of Life questionnaire
EORTC-QLQ-PR25: European Organisation for Research and Treatment of Cancer Core Prostate Module
EPIC: Expanded Prostate Cancer Index
EPR: Electronic patient records
ePRO: Electric patient-reported outcome
ePROMs: Electronic patient-reported outcome measures
EQ-5D: Standardised instrument for use as a measure of health status
eRAPID: Electronic patient self-Reporting of Adverse-events: Patient Information and aDvice
FACT-G: Functional Assessment in Cancer Therapy Scale-General
FDA: Federal Drug Agency
FPQ: Female pelvic questionnaire (questionnaire developed at The Christie Manchester to monitor patients receiving RT to the pelvis)
GCP: Good Clinical Practice
HEAP: Health Economics Analysis Plan
IMRT: Intensity-modulated radiation therapy an advanced high-precision mode using linear accelerators
ISAAC: Online system for Interactive Symptom Assessment and Collection
LENT-SOMA: Late effects Normal Tissue-Subjective, Objective, Management, analytic scales
LTHT: Leeds Teaching Hospitals NHS Trust
MPQ: Male pelvic questionnaire (questionnaire developed at The Christie Manchester to monitor patients receiving RT to the pelvis)
MRC: Medical research council
NCEPOD: National Confidential Enquiry into Patient Outcome and Death
NHS: National Health Service
NIHR: National Institute for Health Research
OST: Observational and supportive team (The Christie)
PAM: The Patient Activation Measure (tool for measuring the level of patient engagement in their healthcare)
PPM: Patient Pathway Manager (electronic patient record system used in Leeds Teaching Hospitals)
PRO: Patient-reported outcomes
PRO-CTCAE: Patient-Reported Outcomes version of the Common Terminology Criteria for Adverse Events
PROMs: Patient-reported outcome measures
QOL: Quality of life
QTool: Web-based questionnaire builder system (built by X-Lab)
RCT: Randomised Controlled Trial
RT: Radiotherapy
RTOG: Radiation therapy oncology group (toxicity criteria)
SAS: Statistical Analysis Software
STAR: Symptom Tracking and Symptom Reporting
TMG: Trial Management Group
TSC: Trial Steering Committee
X-Lab: A private software company based in Leeds
