# Supplementary material for: eRAPID electronic patient self-Reporting of Adverse-events: Patient Information and aDvice: a pilot study protocol in pelvic radiotherapy
Source: Pilot Feasibility Stud. 2018 Jun 5;4:110. doi: 10.1186/s40814-018-0304-6 (PMC5987546; doi:10.1186/s40814-018-0304-6)
Supplement: Supplementary file 1 — Appendix 1. Radiotherapy eRAPID symptom report items. Appendix 2. Patient information sheet. Appendix 3. Patient consent form. (DOCX 50 kb) [file 40814_2018_304_MOESM1_ESM.docx]

## Appendix 1

| Radiotherapy eRAPID symptom report items | | | | | | |
| --- | --- | --- | --- | --- | --- | --- |
| KEY: Cancer groups: PR=Prostate, GY=Cervical / Vaginal / Endometrial / Vulval, AR=Anorectal  Symptom grading: 1=Level 1, 2=Level 2, 3=Level 3 | | | | | | |
| Item | Standard or Dropdown | Responses | **PR** | **GY** | | **AR** |
| **NON-STOMA BOWEL QUESTIONS** | | | | | | |
|  |  |  |  |  | |  |
| To what extent have you had pain in your lower tummy? | Standard | None at all | 0 | 0 | | 0 |
|  |  | Minimal | 1 | 1 | | 1 |
|  |  | Tolerable with painkillers (but does not interfere with activities) | 2 | 2 | | 2 |
|  |  | Intense, requires painkillers (interferes with activities) | 3 | 3 | | 2 |
|  |  | Excruciating (interferes with all activities) | 3 | 3 | | 3 |
|  |  |  |  |  | |  |
| To what extent have you had pain when you open your bowels? | Standard | None at all | 0 | 0 | | 0 |
|  |  | Minimal | 1 | 1 | | 1 |
|  |  | Tolerable with painkillers (but does not interfere with activities) | 2 | 1 | | 2 |
|  |  | Intense, requires painkillers (interferes with activities) | 3 | 2 | | 2 |
|  |  | Excruciating (interferes with all activities) | 3 | 3 | | 2 |
|  |  |  |  |  | |  |
| Have you had any diarrhoea? | Standard | No | 0 | 0 | | 0 |
|  |  | Same occurrence as usual | 0 | 0 | | 0 |
|  |  | Increase of up to 4-times daily | 1 | 1 | | 1 |
|  |  | Increase of up to 4-6 times daily | 2 | 2^a^ | | 2 |
|  |  | Increase of 7 times plus daily | 3 | 3 | | 3 |
|  |  |  |  |  | |  |
| Have you had constipation? | Standard | No I have emptied my bowels more than four times a week | 0 | 0 | | 0 |
|  |  | I have emptied my bowels 3-4 times a week | 0 | 0 | | 0 |
|  |  | I have emptied my bowels 2 times a week | 1 | 1 | | 1 |
|  |  | I have emptied my bowels once a week | 2 | 2^a^ | | 2 |
|  |  | I have emptied my bowels less than once a week | 3 | 3 | | 3 |
|  |  |  |  |  | |  |
| When you feel a desire to open your bowels, how often do you need to use the toilet straight away (urgently)? | Standard | Never | 0 | 0 | | 0 |
|  |  | Monthly or less | 0 | 0 | | 0 |
|  |  | Weekly | 1 | 1 | | 1 |
|  |  | Daily | 2* | 2 | | 1 |
|  |  | Constantly | 2* | 3 | | 2 |
|  |  |  |  |  | |  |
| When you feel a need to open your bowels urgently, how often were you unable to do so? | Standard | Never | 0 | 0 | | 0 |
|  |  | Monthly or less | 0 | 0 | | 0 |
|  |  | Weekly | 1 | 1 | | 1 |
|  |  | Daily | 2 | 2 | | 2 |
|  |  | Constantly | 2 | 2 | | 2 |
|  |  |  |  |  | |  |
| Have you had any difficulty controlling your bowels (e.g. leaked stools)? | Standard | No | 0 | 0 | | 0 |
|  |  | Yes, monthly | 1 | 1 | | 0 |
|  |  | Yes, weekly | 2 | 2 | | 2 |
|  |  | Yes, daily | 3 | 2 | | 2 |
|  |  | Yes, constantly | 3 | 2 | | 3 |
| **URINARY QUESTIONS** | | | | | | |
|  |  |  |  |  | |  |
| Do you use a urinary catheter? (tube inserted to drain urine from the bladder). | Standard | No | 0 |  | |  |
|  |  | Yes | 0 |  | |  |
|  |  |  |  |  | |  |
| To what extent have you had pain when you pass urine? | Standard | None at all | 0 | 0 | | 0 |
|  |  | Minimal | 1 | 0 | | 0 |
|  |  | Tolerable with painkillers (but does not interfere with activities) | 2 | 1 | | 1 |
|  |  | Intense, requires painkillers (interferes with activities) | 2* | 2 | | 2 |
|  |  | Excruciating (interferes with all activities) | 2* | 3 | | 2 |
|  |  |  |  |  | |  |
| When you feel a desire to pass urine, how often do you need to use the toilet straight away (urgently)? | Standard | Never | 0 | 0 | | 0 |
|  |  | Monthly or less | 0 | 0 | | 0 |
|  |  | Weekly | 1 | 1 | | 1 |
|  |  | Daily | 2* | 2 | | 1 |
|  |  | Constantly | 2* | 3 | | 2 |
|  |  |  |  |  | |  |
| How frequently have you passed urine? | Standard | Less than normal | 0 | 0 | | 0 |
|  |  | Normally | 0 | 0 | | 0 |
|  |  | More often than normal, but it does not interfere with daily activities | 1 | 1 | | 1 |
|  |  | More often than normal, and it does interfere with daily activities | 2 | 2 | | 2 |
|  |  |  |  |  | |  |
| Has your flow of urine changed? | Standard | No | 0 |  | | 0 |
|  |  | Yes, I have occasional difficulty with either starting to urinate, taking a long time to urinate, or with emptying my bladder | 1 |  | | 1 |
|  |  | Yes, I have difficulty about half the time with either starting to urinate, taking a long time to urinate, or with emptying my bladder | 2 |  | | 2 |
|  |  | Yes, I have difficulty almost all the time with either starting to urinate, taking a long time to urinate, or with emptying my bladder | 2^†^ |  | | 2^†^ |
|  |  |  |  |  | |  |
| Are you currently unable to urinate? | ^†^Branching | I am currently able to urinate | 2 |  | | 2 |
|  |  | I am currently able to urinate because it is managed by catheter/medication | 2 |  | | 2 |
|  |  | I am currently unable to urinate, and it is not managed by catheter/medication | 3 |  | | 3 |
|  |  |  |  |  | |  |
| Have you had any difficulty controlling your bladder (e.g. leaked urine)? | Standard | No | 0 | 0 | | 0 |
|  |  | Yes, monthly | 1 | 1 | | 1 |
|  |  | Yes, weekly | 2 | 2 | | 2 |
|  |  | Yes, daily | 3 | 2 | | 2 |
|  |  | Yes, constantly | 3 | 2 | | 3 |
|  |  |  |  |  | |  |
| Do you have to get up in the night to pass urine? | Standard | No | 0 |  | 0 | |
|  |  | Yes, but only once | 0 |  | 1 | |
|  |  | 2-3 times | 1 |  | 1 | |
|  |  | 4-6 times | 2 |  | 2 | |
|  |  | 7 plus times | 3 |  | 2 | |
|  |  |  |  |  | |  |
| Have you had any sore skin around your anal area? | Standard | Not at all |  |  | | 0 |
|  |  | A little |  |  | | 1 |
|  |  | Quite a bit |  |  | | 2 |
|  |  | Very much |  |  | | 2 |
|  |  |  |  |  | |  |
| Have you had any changes to your skin in the area treated with radiotherapy? | Standard | No |  |  | | 0 |
|  |  | My skin is a faint pink-red colour or is dry and flaking, but not too uncomfortable |  |  | | 1 |
|  |  | My skin is a pink-red colour, is swollen or has some patches which are moist/weeping (mostly within skin folds and creases), and is uncomfortable or sore |  |  | | 2 |
|  |  | My skin has wide areas which are moist/weeping or bleeding and is very sore or uncomfortable |  |  | | 2^†^ |
|  |  |  |  |  | |  |
| Is this a new problem and are your medical team aware of it? | ^†^Branching | This is a new problem and/or my medical team don't know about it |  |  | | 3 |
|  |  | This is an existing problem and my medical team know about it |  |  | | 2 |
|  |  |  |  |  | |  |
| Have you had any blood in your urine? | Standard | None | 0 | 0 | | 0 |
|  |  | Yes, a little blood in urine compared to what is normal for me | 3 | 3 | | 1 |
|  |  | Yes, a lot of blood in urine compared to what is normal for me | 3 | 3 | | 2 |
|  |  |  |  |  | |  |
| Have you had any bleeding when you open your bowels? | Standard | None | 0 | 0 | | 0 |
|  |  | Yes, a little bleeding when opened bowels compared to what is normal for me | 3 | 3 | | 1 |
|  |  | Yes, a lot of bleeding when opened bowels compared to what is normal for me | 3 | 3 | | 2 |
|  |  |  |  |  | |  |
| Have you had any vaginal bleeding? | Standard | None |  | 0 | | 0 |
|  |  | Yes, a little vaginal bleeding compared to what is normal for me |  | 3 | | 1 |
|  |  | Yes, a lot of vaginal bleeding compared to what is normal for me |  | 3 | | 2 |
|  |  |  |  |  | |  |
| **SEXUAL QUESTIONS** | | | | | | |
|  |  |  |  |  | |  |
| Do you have any vaginal discharge? | Standard | No |  | 0 | | 0 |
|  |  | Yes, but only mildly |  | 1 | | 1 |
|  |  | Moderate or heavy enough to need pads |  | 2 | | 2 |
|  |  |  |  |  | |  |
| Has your treatment affected your ability to have a sexual relationship? | Standard | No | 0 | 0 | | 0 |
|  |  | Not in a sexual relationship |  | 0 | | 0 |
|  |  | Yes, but not a problem | 0 | 0 | | 1 |
|  |  | Yes, and it is a problem | 2^†^ | 0^†^ | | 2 |
|  |  | Not attempted intercourse |  | 0 | | 0 |
|  |  | Not applicable | 0 |  | | 0 |
|  |  | Prefer not to answer | 0 | 0 | | 0 |
|  |  |  |  |  | |  |
| How would you describe the usual quality of your erections? | ^†^Branching | Firm enough for intercourse | 0 |  | | 0 |
|  |  | Firm enough for masturbation and foreplay | 1 |  | | 1 |
|  |  | Not firm enough for any sexual activity | 2 |  | | 2 |
|  |  | None at all | 2 |  | | 2 |
|  |  | Not applicable | 0 |  | | 0 |
|  |  | Prefer not to answer | 0 |  | | 0 |
|  |  |  |  |  | |  |
| How would you rate your ability to reach orgasm (climax)? | Standard | Very good | 0 |  | | 0 |
|  |  | Good | 0 |  | | 0 |
|  |  | Fair | 1 |  | | 1 |
|  |  | Poor | 2 |  | | 2 |
|  |  | Very poor to none | 2 |  | | 2 |
|  |  | Not applicable | 0 |  | | 0 |
|  |  | Prefer not to answer | 0 |  | | 0 |
|  |  |  |  |  | |  |
| Have you felt less masculine as a result of your illness? | Standard | Not at all | 0 |  | | 0 |
|  |  | A little | 1 |  | | 1 |
|  |  | Quite a bit | 2 |  | | 2 |
|  |  | Very much | 2 |  | | 2 |
|  |  | Prefer not to answer | 0 |  | | 0 |
|  |  |  |  |  | |  |
| Have you been given a vaginal dilator? | Standard | Yes, and I know how to use it |  | 0 | | 0 |
|  |  | Yes, but I am not confident with using it |  | 2 | | 2 |
|  |  | No, but I need one |  | 0 | | 0 |
|  |  | I don't need one |  | 0 | | 0 |
|  |  | Prefer not to answer |  | 0 | | 0 |
|  |  |  |  |  | |  |
| Do you have vaginal dryness? | Standard | No |  | 0 | | 0 |
|  |  | Yes, but only mildly |  | 1 | | 1 |
|  |  | Yes, and interferes with intercourse/using dilator |  | 1 | | 2 |
|  |  | Yes, and it is severe enough to stop intercourse/using dilator |  | 2 | | 2 |
|  |  | Not applicable |  | 0 | | 0 |
|  |  | Prefer not to answer |  | 0 | | 0 |
|  |  |  |  |  | |  |
| Has your vagina felt tight or short? | Standard | No |  | 0 | | 0 |
|  |  | Yes, but only mildly |  | 1 | | 1 |
|  |  | Yes, and interferes with intercourse/using dilator |  | 1 | | 2 |
|  |  | Yes, and it is severe enough to stop intercourse/using dilator |  | 2 | | 2 |
|  |  | Not applicable |  | 0 | | 0 |
|  |  | Prefer not to answer |  | 0 | | 0 |
|  |  |  |  |  | |  |
| Have you experienced pain with intercourse? | Standard | No |  | 0 | | 0 |
|  |  | Yes, but only mildly |  | 1 | | 1 |
|  |  | Yes, and interferes with intercourse/using dilator |  | 1 | | 2 |
|  |  | Yes, and it is severe enough to stop intercourse/using dilator |  | 2 | | 2 |
|  |  | Not applicable |  | 0 | | 0 |
|  |  | Prefer not to answer |  | 0 | | 0 |
|  |  |  |  |  | |  |
| **Are you taking tablets/medication for any of the following?** | Standard | Diarrhoea | 0 | 0 | | 0 |
|  |  | Constipation | 0 | 0 | | 0 |
|  |  | For bladder | 0 | 0 | | 0 |
|  |  | For pain |  | 0 | | 0 |
|  |  | For erection problems | 0 |  | |  |
|  |  | To block male hormones | 0 |  | |  |
| **FATIGUE AND PHYSICAL QUESTIONS** | | | | | | |
|  |  |  |  |  | |  |
| Overall, how big a problem has lack of energy been for you? | Standard | No problem | 0 | 0 | |  |
|  |  | Very small problem | 0 | 0 | |  |
|  |  | Small problem | 1 | 1 | |  |
|  |  | Moderate problem | 2 | 2 | |  |
|  |  | Big problem | 2 | 2 | |  |
|  |  |  |  |  | |  |
| Overall, how big a problem has lack of energy been for you? | Standard | No problem |  |  | | 0 |
|  |  | I felt tired but this was relieved by rest |  |  | | 1 |
|  |  | I felt tired but this was not relieved by rest and I had difficulty doing some of the things I normally do (e.g. household chores, shopping) |  |  | | 1 |
|  |  | I felt tired but this was not relieved by rest and I was not able to take care of myself (e.g. getting out of bed, bathing, dressing) |  |  | | 2 |
|  |  |  |  |  | |  |
| Please select from the options below the statement that best describes your level of physical ability during the past week | Standard | I am up and about and can do everything that I could do before my illness without any difficulty | 0 | 0 | | 0 |
|  |  | I am up and about almost all of the time. I cannot do any heavy physical work but I can do most other things (e.g. household chores, shopping) | 1 | 1 | | 1 |
|  |  | I am up and about more than half the day; I can look after myself (e.g. getting out of bed, bathing, dressing) but I cannot do much else | 2 | 2 | | 2 |
|  |  | I am in bed or in a chair for more than half of the day and I need some help looking after myself | 2 | 2 | | 2 |
|  |  | I am in bed or in a chair all the time and I cannot look after myself | 2^†^ | 2^†^ | | 3 |
|  |  |  |  |  | |  |
| Is there someone with you who can help you take care of yourself? | ^†^Branching | No, there is no one with me who can help me | 3 | 3 | |  |
|  |  | Yes, there is someone here who can help me | 2 | 2 | |  |
| **CHEMOTHERAPY QUESTIONS** | | | | | | |
|  |  |  |  |  | |  |
| Have you had pain or discomfort anywhere on your body? | Standard | No |  | 0 | | 0 |
|  |  | I had mild pain or discomfort |  | 1 | | 1 |
|  |  | I had moderate pain or discomfort and I was not able to do some of the things I normally do (e.g. household chores, shopping) |  | 2 | | 2 |
|  |  | I had severe pain or discomfort and I was not able to care for myself (e.g. getting out of bed, bathing, dressing) |  | 3 | | 3 |
|  |  |  |  |  | |  |
| Have you felt sick (nauseous/queasy)? | Standard | No |  | 0 | | 0 |
|  |  | I felt sick but I was able to eat and drink the same amount and type of foods as usual |  | 1 | | 1 |
|  |  | I felt sick and I ate or drank less than usual or changed what I ate or drank |  | 2 | | 2 |
|  |  | I felt sick and was not able to eat or drink |  | 3 | | 3 |
|  |  |  |  |  | |  |
| Have you been sick (vomited)? | Standard | No |  | 0 | | 0 |
|  |  | I have vomited 1 - 2 times in a 24 hour period |  | 1 | | 1 |
|  |  | I have vomited 3 - 5 times in a 24 hour period |  | 2 | | 2 |
|  |  | I have vomited 6 or more times in a 24 hour period |  | 3 | | 3 |
|  |  |  |  |  | |  |
| Please take your temperature with a thermometer and choose the best answer below. | Standard | My temperature is less than 37.5 ⁰C |  |  | | 0 |
|  |  | My temperature is between 37.5 ⁰C and 37.9 ⁰C |  |  | | 2 |
|  |  | My temperature is between 38 ⁰C and 38.4 ⁰C |  |  | | 3 |
|  |  | My temperature is 38.5 ⁰C or above |  |  | | 3 |
|  |  |  |  |  | |  |
| Please take your temperature with a thermometer and choose the best answer below. | Standard | My temperature is less than 36°C |  | 3 | |  |
|  |  | My temperature is between 36°C and 37°C |  | 0 | |  |
|  |  | My temperature is between 37.1°C and 37.4°C |  | 2 | |  |
|  |  | My temperature is 37.5°C or above |  | 3 | |  |
|  |  |  |  |  | |  |
| Have you had shivering, shaking and chattering of teeth? | Standard | No |  | 0 | | 0 |
|  |  | I have felt a little cold with shivering and chattering of teeth |  | 2 | | 1 |
|  |  | I have felt very cold, with shaking all over my body but this settled very quickly |  | 2 | | 2 |
|  |  | I have felt very cold, with shaking all over my body for 30 minutes or more |  | 3 | | 3 |
|  |  |  |  |  | |  |
| Have you lacked appetite or lost interest in food? | Standard | No |  | 0 | | 0 |
|  |  | I have lacked appetite but I was able to eat and drink the SAME AMOUNT and type of food as usual |  | 1 | | 1 |
|  |  | I have lacked appetite and I ate or drank LESS THAN usual or I have been taking supplement drinks |  | 2 | | 2 |
|  |  | I have lacked appetite and I was not able to eat or drink |  | 3 | | 3 |
|  | | | | | | |
| Have you had a sore mouth or tongue? | Standard | No |  |  | | 0 |
|  |  | My mouth was a bit sore |  |  | | 1 |
|  |  | My mouth was quite sore but I was still able to eat soft foods and drink fluids |  |  | | 2 |
|  |  | My mouth was very sore and I was not able to eat or drink |  |  | | 3 |
|  |  |  |  |  | |  |
| Have you had soreness or redness of your hands and feet | Standard | None |  |  | | 0 |
|  |  | My skin was a bit red but was not painful |  |  | | 1 |
|  |  | My skin was painful, red, cracked or peeling and I was not able to do some of the things I normally do (e.g. household chores, shopping) |  |  | | 2 |
|  |  | My skin was very painful and I was not able to care for myself (e.g. getting out of bed, bathing, dressing) |  |  | | 3 |
| **STOMA BOWEL QUESTIONS** | | | | | | |
|  | | | | | | |
| Do you have a stoma (colostomy bag) or ileostomy? | Standard | Yes |  |  | | 0 |
|  |  | No |  |  | | 0 |
|  |  |  |  |  | |  |
| Have you increased the number of times you have had to empty your stoma bag? | Standard | No |  |  | | 0 |
|  |  | I’m emptying my stoma bag 2 - 3 times more in a 24 hour period than is normal for me |  |  | | 1 |
|  |  | I’m emptying my stoma bag 4 - 6 times more in a 24 hour period than is normal for me |  |  | | 2 |
|  |  | I’m emptying my stoma bag over 7 times more in a 24 hour period than is normal for me |  |  | | 3 |
|  |  |  |  |  | |  |
| Have you had constipation recently? | Standard | No, I’m emptying my stoma bag more than 4 times a week |  |  | | 0 |
|  |  | I’m emptying my stoma bag 3-4 times a week |  |  | | 0 |
|  |  | I’m emptying my stoma bag 2 times a week |  |  | | 1 |
|  |  | I’m emptying my stoma bag once a week |  |  | | 2 |
|  |  | I’m emptying my stoma bag less than once a week |  |  | | 3 |
|  |  |  |  |  | |  |
| Have you had any sore skin around your stoma? | Standard | Not at all |  |  | | 0 |
|  |  | A little |  |  | | 1 |
|  |  | Quite a bit |  |  | | 2 |
|  |  | Very much |  |  | | 2 |
|  |  |  |  |  | |  |
| Did you feel embarrassed because of your stoma? | Standard | Not at all |  |  | | 0 |
|  |  | A little |  |  | | 1 |
|  |  | Quite a bit |  |  | | 2 |
|  |  | Very much |  |  | | 2 |
|  |  |  |  |  | |  |
| Has your stoma bag burst or come off/dislodged? | Standard | Not at all |  |  | | 0 |
|  |  | Yes, occasionally |  |  | | 1 |
|  |  | Yes, on a daily basis |  |  | | 2 |
|  |  |  |  |  | |  |
| Have you had unintentional release of gas/flatulence from your stoma? | Standard | No |  |  | | 0 |
|  |  | A little |  |  | | 1 |
|  |  | Quite a bit |  |  | | 1 |
|  |  | Very much |  |  | | 2 |
| **DROPDOWN QUESTIONS** | | | | | | |
|  |  |  |  |  | |  |
| Has your physical condition or medical treatment interfered with your family life? | Dropdown | No | 0 | 0 | | 0 |
|  |  | A little | 1 | 1 | | 1 |
|  |  | Quite a bit | 2 | 2 | | 1 |
|  |  | Very much | 2 | 2 | | 2 |
|  |  |  |  |  | |  |
| Has your physical condition or medical treatment interfered with your social activities? | Dropdown | No | 0 | 0 | | 0 |
|  |  | A little | 1 | 1 | | 1 |
|  |  | Quite a bit | 2 | 2 | | 1 |
|  |  | Very much | 2 | 2 | | 2 |
|  |  |  |  |  | |  |
| Has weight gain been a problem for you? | Dropdown | No | 0 |  | |  |
|  |  | A little | 1 |  | |  |
|  |  | Quite a bit | 2 |  | |  |
|  |  | Very much | 2 |  | |  |
|  |  |  |  |  | |  |
| Did you have hot flushes? | Dropdown | No | 0 | 0 | | 0 |
|  |  | A little | 1 | 1 | | 1 |
|  |  | Quite a bit | 2 | 1 | | 1 |
|  |  | Very much | 2 | 2 | | 2 |
|  |  |  |  |  | |  |
| Have you had sore or enlarged nipples or breasts? | Dropdown | No | 0 |  | |  |
|  |  | A little | 1 |  | |  |
|  |  | Quite a bit | 2 |  | |  |
|  |  | Very much | 2 |  | |  |
|  |  |  |  |  | |  |
| Have you had unintentional release of gas/flatulence from your back passage? | Dropdown | A little |  | 1 | | 1 |
|  |  | Quite a bit |  | 1 | | 1 |
|  |  | Very much |  | 2 | | 2 |
|  |  |  |  |  | |  |
| Have you ever experienced feelings of anxiety (for example feelings of dread, restlessness, panic and tension)? | PR=  Dropdown  GY=  Standard | None | 0 | 0 | | 0 |
|  |  | I occasionally have experienced some of these feelings but it passed or I was able to calm myself down | 1 | 1 | | 1 |
|  |  | I have quite often experienced these feelings and they have interfered with my daily activities (e.g. housework/shopping) | 2 | 2 | | 1 |
|  |  | I have frequently experienced these feelings and I am finding it difficult to care for myself (e.g. bathing, getting dressed) | 2 | 3 | | 2 |
|  |  |  |  |  | |  |
| Have you felt low in mood (e.g. hopeless, helpless, irritable, guilty or worthless)? | PR=  Dropdown  GY=  Standard | None | 0 | 0 | | 0 |
|  |  | I occasionally felt low in mood but it passed or I could lift myself out of it | 1 | 1 | | 1 |
|  |  | I quite often feel low in mood and no longer enjoy things I used to and find it difficult to carry out daily activities (e.g. household chores, shopping) | 2 | 2 | | 2 |
|  |  | I have frequently experienced these feelings and I am finding it difficult to care for myself (e.g. getting out of bed, bathing, dressing) | 2 | 3 | | 2 |
|  |  |  |  |  | |  |
| Have you had difficulty sleeping? | Dropdown | No | 0 | 0 | | 0 |
|  |  | I occasionally have difficulty falling asleep, staying asleep or I wake too early | 1 | 1 | | 1 |
|  |  | I often have difficulty falling asleep, staying asleep or I wake too early | 2 | 2 | | 2 |
|  |  | I always have difficulty falling asleep, staying asleep or I wake too early | 2 | 2 | | 2 |
|  |  |  |  |  | |  |
| Have you had any tingling or numbness in your fingers or toes? | Dropdown  AR=  Standard | No |  | 0 | | 0 |
|  |  | I had a bit of tingling or numbness (perhaps when handling cold or hot objects) |  | 1 | | 1 |
|  |  | I had tingling or numbness and I was not able to do some of the things I normally do (e.g. buttoning up or using cutlery) |  | 1 | | 2 |
|  |  | I had tingling or numbness and I was not able to carry out daily activities (e.g. I had difficulty walking, dropped things or stepped on things by accident) |  | 2 | | 2 |
|  |  |  |  |  | |  |
| What was the severity of your indigestion or heartburn? | Dropdown | None |  | 0 | | 0 |
|  |  | Mild |  | 1 | | 1 |
|  |  | Moderate |  | 2 | | 2 |
|  |  | Severe |  | 3 | | 3 |
|  |  | Very severe |  | 3 | | 3 |
|  |  |  |  |  | |  |

## Appendix 2

## Patient Information Sheet

**eRAPID E**lectronic patient self-**R**eporting of **A**dverse-events: **P**atient **I**nformation and a**D**vice: **Feasibility pilot study in radiotherapy**

We would like to invite you to take part in a research study that will assess a new online system for monitoring the symptoms and side effects cancer patients can experience when receiving treatment.

Before you decide whether to take part, please read this information sheet to find out why the research is being done and what it involves. Please take time to read the information carefully. Talk to others about the study if you wish, and ask the researcher if you have any questions.

**Background**

This project is called eRAPID. This stands for “Electronic patient self-Reporting of Adverse-events: Patient Information and aDvice”. eRAPID is an online system for you to report symptoms and side effects during and after cancer treatment. This information is, in a timely way (within 5 minutes), transferred and documented in your individual electronic patient record in the hospital. If you report mild side effects, you will receive advice on how to manage them and how to seek medical advice.

So far during the eRAPID project we have developed the web-based system, called QTool, for patients to report symptoms and side effects of cancer and we have established the real time transfer of the data to the hospital records. We have also designed a website giving advice on managing symptoms and to help live with cancer.

We want to see how the system will work in practice in a large-scale study – for example whether patients use the system and their experiences of using it to report their symptoms. We also want staff involved in patient care to test the system by viewing the results of the questionnaires in patient records and to tell us how useful the information is. We will be assessing how the system impacts on patient care and quality of life.

The study is part of a 5-year research programme funded by the National Institute for Health Research (NIHR). The research is led by Professor Galina Velikova who is a consultant medical oncologist with Leeds Teaching Hospitals NHS Trust.

**Why have I been asked?**

We will be asking 84 patients who are currently receiving treatment for prostate, anal, rectal, vaginal or cervical cancer at St James’s University Hospital to take part to evaluate how the system could potentially work with real patients in a clinic setting.

**Do I have to take part?**

No. It is up to you to decide whether or not to take part. We are interested in understanding why people do not wish to take part in the study but you do not have to give us a reason for doing so. A decision not to take part will not affect the standard of care or treatment you receive in the future.

**What will happen if I take part in the study?**

You can take as much time as you need to decide if you want to take part in the study or not. If you decide to take part in the study, a member of the research team will answer any questions you have and ask you to sign a consent form.

You will then be asked to complete some initial paper questionnaires about you and your quality of life. This study is a randomised controlled trial (RCT). This means half the participants who agree to help with the study will be asked to use the eRAPID symptom reporting system and the eRAPID website during the study (in addition to Usual Care from the hospital and cancer team) and the other half will receive Usual Care alone. This way we can compare the two groups to see if the eRAPID system has any impact on patient care. Participant allocation to one of the groups is entirely by chance.

If you are assigned to the group using the eRAPID system:

- The researcher will explain how to complete the online eRAPID symptom questionnaire and show you a brief demonstration of the system at a routine hospital visit.
- You will be given unique log-in details and a booklet to take home with you that explains how to use the system. You are welcome to ask a friend or family member to help you access the online system if this would be helpful, though we would like you to answer the symptom questions yourself.
- Whilst you are on treatment you will receive the usual care provided by the hospital and your cancer team but in addition you will be asked to log-in to the eRAPID system from home at least once a week to complete the symptom questionnaire. We will also encourage you to complete the questionnaire at any time when you feel unwell. The questionnaire takes around 15 minutes to complete. Your responses will be immediately documented in your electronic patient records.
- We will automatically send you a reminder each week to complete the questionnaire by your choice of email or text message.
- There may also be the opportunity to complete the questionnaire in clinic before routine appointments.
- We will also ask you at set time points (6, 12 and 24 weeks after you join the study) to complete some paper questionnaires about your quality of life and views of your health and treatment. We will also ask you to tell us about the number of contacts you have had with the hospital and GP while you are helping with the study (e.g. appointments and telephone calls). We are interested in understanding any extra financial costs you may have experienced as the result of receiving cancer treatment so the questionnaires will ask you about this too.
- At the end of the study, we are asking a proportion of patients (up to 5 per disease group) to take part in an end of study interview or to complete an end of study feedback form to find out what you thought about the research and the eRAPID system. The interviews will take place in a private room within a clinical area at the hospital. These interviews will be audio recorded and last approximately 30 minutes. Anonymised extracts from these interviews may be used in reports, publications or presentations.
- The eRAPID system is not a replacement for usual care. If you need advice on managing any symptoms or side effects, you will still be advised to contact the hospital staff.

If you are assigned to the Usual Care group:

- You will receive the usual care provided by the hospital and your cancer team.

In addition, the researchers will ask you at set time points (6, 12 and 24 weeks after you join the study) to complete paper questionnaires about your quality of life and views of your health and treatment. We will also ask you to tell us about the number of contacts you have had with the hospital and GP (appointments and telephone calls) while you are helping with the study. We are interested in understanding any extra financial costs you may have experienced as the result of receiving cancer treatment so the questionnaires will ask you about this too.

**Will my taking part in this study be kept confidential?**

Yes. It is very important to us to respect your information (data) and keep it confidential. The answers you provide to the symptom and side effect questions, and additional questionnaires will only be seen by the research team and your clinical team. We will also ask for your permission to look at your medical records through the Patient Pathway Management (PPM) system for information about the treatment you are receiving, disease condition, clinical care, hospital resource and management records.

All your data (questionnaires/audio recorded interviews) will be stored on secure databases within either the University of Leeds or Leeds Teaching Hospitals NHS Trust or in locked filing cabinets and will only be accessible by the research and clinical teams. All data stored on the University of Leeds databases will be anonymised. All information from audio-recorded interviews are completely confidential. The interview recording will be downloaded from the recorder and kept securely on University of Leeds computers and will only be available to the research team. After your interview, we will type up a transcript of what was said. The person typing this up will take out all names and identifying information. The transcript will be kept separate from your personal details, and will only be linked to you using a code. Only the researchers will have access to that code.

All data will be securely archived at the University of Leeds for 5 years, after which it will be confidentially destroyed. Any analysis or publications of results from the study will not name or identify any individual patients.

**What are the disadvantages of taking part?**

We do not foresee any disadvantages to your taking part in the study.

**What are the possible benefits of taking part?**

We hope that the completion of the online eRAPID questionnaires will help patients and staff with monitoring and managing symptoms and side effects of cancer treatment.

For participants in the Usual Care group, although there may be no personal benefits to your taking part in this study, we hope the information you provide will contribute to improving the support we can offer patients on cancer treatment in the future.

**What will happen if I don’t want to carry on with the study?**

If you agree to take part and then later decide you want to stop being in the study that is OK. You can withdraw from the study at any time. We will ask you if we may keep the information you have provided up until that point but this will be your decision. If you withdraw from the study we may ask you to complete a brief end of study feedback form to find out what you thought about the study and using the eRAPID system for reporting side effects. Again, it will be your decision whether you wish to provide this feedback.

**What if there is a problem?**

We have an established procedure for dealing with patients who are distressed. If distress is detected, the researcher will inform a relevant member of the clinical team (clinic nurses, clinical nurse specialists, radiographers or doctors) who are directly involved with your care, having obtained permission from you first.

If you have any concerns about any aspect of the study please speak to the researchers who will do their best to answer your questions. Their contact details are at the end of this information sheet. If you remain unhappy you may wish to contact the Leeds Teaching Hospitals NHS Trust’s Patient Advice and Liaison Service (PALS)

T: 0113 2066261 or 0113 2067168

E: [patient.relations@leedsth.nhs.uk](mailto:patient.relations@leedsth.nhs.uk)

**Who has reviewed the study?**

The study has been reviewed by people who have experience of cancer themselves and independent experts in this area of research. All research in the NHS is also approved by a Research Ethics Committee, an independent group that works to protect your interests. This study has been reviewed and given favourable opinion by a local Yorkshire and Humber Research Ethics Committee.

**What now?**

Please let the researcher know whether or not you would like to help with the study or if you have any further questions they will be happy to help answer them. If you need more time to think about taking part in the study just let the researcher know and they can speak to you at your next hospital appointment.

**Important**

We are asking you to help us test a new system that is in development and the information that you see online is still undergoing review.

Although doctors or nurses responsible for your care will view the questionnaire information it will only supplement their clinical assessment.

**Taking part in this evaluation will not impact on your routine care, you will still be receiving standard care throughout.**

**This system is not intended to replace any information or advice you have already received.**

**Please continue to report any symptoms, side effects or concerns to your clinical team as you have been advised to do.**

**Thank you for taking the time to read this information sheet.**

If you would like to take part or have any questions please contact:

Simon Pini 0113 206 8952

Andrea Gibson 0113 206 7532

Beverly Clayton 0113 206 7644

E-mail: [leedsth-tr.erapid@nhs.net](mailto:leedsth-tr.erapid@nhs.net)

## Appendix 3

## Patient Consent Form

**eRAPID Electronic patient self-Reporting of Adverse-events: Patient Information and aDvice: Feasibility pilot study in radiotherapy.**

Please **initial** the statements below to show that you have read and agree to each one:

|  | | | | | **Please initial** |
| --- | --- | --- | --- | --- | --- |
| I confirm that I have read and understood the information sheet dated June 2017 Version 1.2 for the above feasibility study. I have had the opportunity to consider the information, ask questions and have had these answered satisfactorily. | | | | | _____________ |
| I understand that my participation is voluntary and that I am free to withdraw at any time without giving any reason, without my medical care or legal rights being affected. | | | | | _____________ |
| I give permission for the research team to store my email address and/or telephone number for the purpose of sending me reminders. | | | | | _____________ |
| I give permission for relevant information from my medical notes and hospital resource and management records to be collected as part of the study by authorised individuals from the research team. | | | | | _____________ |
| I understand that I may be asked to take part in an audio-recorded end of study interview and grant permission for extracts from the written transcripts of the recordings to be used in reports of the research, on the understanding that my anonymity will be maintained. | | | | | _____________ |
| I understand my data will be stored on secure databases within either the University of Leeds or Leeds Teaching Hospitals NHS Trust and will only be accessible by the research and clinical teams. | | | | | _____________ |
| I give permission for my anonymous data to be used for future research studies and/or to be shared with other researchers. | | | | | _____________ |
| I am happy for the contribution I have already made to be used in the analysis if I choose to withdraw from the study. | | | | | _____________ |
| I understand that relevant sections of my medical notes and data collected during the study, may be looked at by individuals from the Patient Reported Outcomes Research Group, from regulatory authorities or from the NHS Trust, where it is relevant to my taking part in this research. I give permission for these individuals to have access to my records. | | | | | _____________ |
| **I agree to take part in the above feasibility pilot study.** | | | | | _____________ |
|  |  |  |  |  | |
| Name of Patient |  | Signature |  | Date | |
|  |  |  |  |  | |
| Name of Researcher |  | Signature |  | Date | |

***When completed: 1 for participant; 1 for researcher site file (original); 1 for medical notes***
